# Relationship between Permeability and Structure of CO_2_-Assisted Polymer Compression Products

**DOI:** 10.3390/membranes13060560

**Published:** 2023-05-29

**Authors:** Takafumi Aizawa

**Affiliations:** Research Institute for Chemical Process Technology, National Institute of Advanced Industrial Science and Technology, 4-2-1 Nigatake, Miyagino-ku, Sendai 983-8551, Japan; t.aizawa@aist.go.jp; Tel.: +81-22-237-5211

**Keywords:** CO_2_-assisted polymer compression, permeability, filter, porosity, tortuosity, pore size, X-ray computed tomography

## Abstract

Membrane filters were fabricated from polyethylene terephthalate nonwoven fabrics with an average fiber diameter of 8 μm using the CO_2_-assisted polymer compression method. The filters were subjected to a liquid permeability test and structural analysis was performed using X-ray computed tomography to evaluate the tortuosity, pore size distribution, and percentage of open pores. Based on the results, filter tortuosity was proposed to be a function of porosity. Pore size estimated from the permeability test and X-ray computed tomography were in rough agreement. The ratio of open pores to all pores was as high as 98.5%, even at a porosity of 0.21. This may be due to the process of exhausting trapped high-pressure CO_2_ after molding. For filter applications, a high open-pore ratio is desirable since it means that many pores are involved in the fluid flow. The CO_2_-assisted polymer compression method was found to be suitable for the production of porous materials for filters.

## 1. Introduction

CO_2_-assisted polymer compression (CAPC) is a method of plasticizing and crimping polymer fibers in the presence of CO_2_ without using heat or adhesives [1]. Experiments using supercritical CO_2_ have shown that CO_2_ specifically dissolves into the polymer [2,3] and impregnates the amorphous region [4,5]. Consequently, the motility of the polymer chain increases, which results in decreases in the glass transition temperature and melting point [6,7]. The degree of crystallinity of a porous polymer has a considerable influence on the Young’s modulus in the presence of CO_2_ [8].

In the CAPC method, fibers are crimped and immobilized in the shape of a mold in the presence of CO_2_. The adhesion mechanism is described as follows. CO_2_ impregnates the polymer, thereby causing it to plasticize. The intersections of the fibers are bonded by pressing the plasticized polymer fibers. The plasticization is then released by venting the CO_2_, and the shape of fibers is fixed. An advantage of this method is that crimping and molding can be performed simultaneously [9]. Moreover, this approach can easily encapsulate drugs and can be applied to controlled-release tablets and reaction cartridges [10,11]. This process is particularly suitable for treating heat-sensitive agents because it does not apply heat. In addition, CO_2_ permeates through the voids between the fibers; thus, even thick samples with many layers can be processed efficiently, which results in high productivity. Nonwoven fabrics are suitable for use as raw fibrous sheets in CAPC because they can be mass-produced and are inexpensive [12]. Nonwoven fabrics are already widely used in filter applications [13].

After lamination and crimping, a fibrous sheet takes the form of a laminate of fibers running in the transverse direction and a membrane filter can be created. Since the CAPC method is a recently developed technique, the characteristics of the product are not fully known. The permeation characteristics of nitrogen gas through filters made via this technique have been reported, and findings show that filter performance varies with making process conditions [14]. Such filters are used in gas and liquid filtration applications and are an indispensable component in chemical plants and daily life [15,16].

For fluid-permeating applications, it is very important that the porous material not only have pores but also that the pores be open. Since crimping is a method of bonding while collapsing voids, there is a possibility that openings will be blocked. If openings are blocked, the membrane performance will be greatly reduced, so it is significant to know the ratio of open pores to all pores. In addition, when considering the flow in these pores, it is rare for the pores to be straight and the tortuosity is very important to material design [17,18]. Therefore, detailed analysis of the three-dimensional (3D) structure of porous materials is essential to elucidate pore structure and tortuosity.

In this study, liquid permeation characteristics and 3D structural observation via X-ray computed tomography (X-ray CT) were obtained for CAPC porous material. The 3D structural analysis included observation of fiber-to-fiber adhesion from the cross section, evaluation of the tortuosity, analysis of pore size distribution, and estimation of the open pores in the total pores. For liquid permeation analysis, the pore diameter was calculated by combining the tortuosity obtained from structural analysis and compared with the pore diameter obtained by X-ray CT. Then, the suitability of CAPC porous materials for fluid permeation applications was discussed based on the percentage of open pores.

## 2. Materials and Methods

### 2.1. Materials

Polyethylene terephthalate (PET) nonwoven fabric with a basis weight of 30 g m^−2^ was used as the raw material. The fabric was made from PET pellets (TK3, Bell Polyester Products Inc., Houfu, Japan) through the melt-blowing method [19] by Nippon Nozzle Co. Ltd. (Kobe, Japan) and its average fiber diameter was adjusted to 8 μm. Samples with a diameter of 18 mm were punched out of the nonwoven using a punch. The number and weight of the sheets were adjusted to match because of the large sample variation due to the nonwoven fabric characteristics. The sets of nonwovens used to make the filters were 10-ply (0.083 g), 12-ply (0.099 g), 14-ply (0.116 g), 16-ply (0.132 g), 18-ply (0.149 g), and 20-ply (0.165 g). The sets of nonwovens for X-ray CT were 8-ply (0.062 g), 10-ply (0.078 g), 12-ply (0.093 g), 14-ply (0.109 g), and 16-ply (0.125 g).

### 2.2. CAPC Process

**Figure 1:**
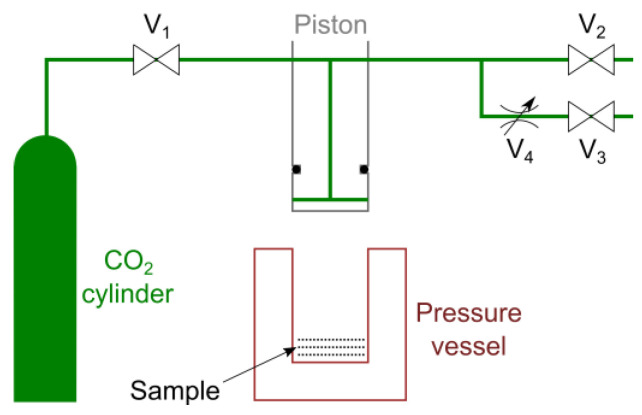
Schematic of the CO_2_-assisted polymer compression (CAPC) equipment. V_1_, CO_2_ introduction valve; V_2_, exhaustion valve for rapid exhaustion; V_3_, exhaustion valve for slow exhaustion; V_4_, metering valve.

A flow diagram of the apparatus used for making CAPC products is shown in Figure 1. CAPC was performed at room temperature (23 °C). A set of raw nonwoven fabrics was placed in a stainless-steel container, which was then placed under a piston. The piston was lowered to the CO_2_ introduction position, and CO_2_ was introduced and exhausted three times to replace the air in the container with CO_2_. After CO_2_ introduction at vapor pressure (6 MPa), the piston was lowered to the press position and held there for 10 s. Afterward, CO_2_ was slowly released into the atmosphere for 30 s by passing it through the V_4_ metering valve and then opening the V_2_ valve. The piston was raised and the sample was fabricated in the container. The filter was adjusted to a thickness of 0.6 mm, and the CO_2_ introduction and press piston positions were 0.9 and 0.6 mm, respectively. Each sample for X-ray CT was adjusted to a thickness of 0.5 mm, and the CO_2_ introduction and press piston positions were 0.75 and 0.5 mm, respectively.

### 2.3. Liquid Permeation Test

**Figure 2:**
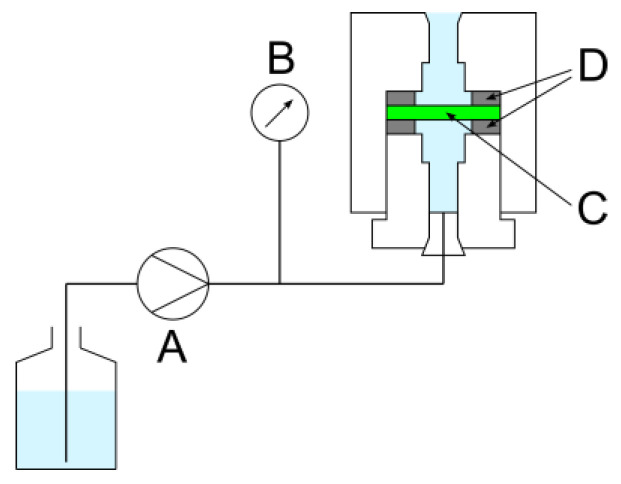
Schematic of ethanol solution permeability measurements. A, pump; B, pressure gauge; C, sample; D, gaskets.

The setup for the ethanol solution permeability measurements is shown in Figure 2. These experiments involved a double-plunger pump for liquid chromatography (Shimadzu Co., Kyoto, Japan; LC-10ADvp) and a digital pressure gauge with Bluetooth output (Krone Co., Katsushika-ku, Japan; KDM30-BT; 35 kPaG). Fluoro rubber gaskets with 8.73 mm diameter holes were used. A 10 wt% ethanol aqueous solution was set in the filter holder and then flowed at the maximum flow rate for at least 30 min. The solution was allowed to overflow from the filter holder, with the outlet facing upward, to eliminate the air bubbles in the filter. The pump was then stopped once, and the pressure gauge was reset to zero. The flow rate was then increased in five steps according to the filter density, and the pressure drop was measured. The pressure was monitored by transferring the pressure measurement to a PC every 2 s. At least 5 min after the pressure stabilized, the flow rate was changed for the next measurement. The flow rate settings were 1, 2, 3, 4, and 5 mL min^−1^ for the 10-ply laminated products; 0.5, 1, 1.5, 2, and 2.5 mL min^−1^ for the 12- and 14-ply laminated products; 0.25, 0.5, 0.75, 1, and 1.25 mL min^−1^ for the 16- and 18-ply laminated products; and 0.1, 0.2, 0.3, 0.4, and 0.5 mL min^−1^ for the 20-ply laminated product. The experiments were performed five times at each flow rate.

### 2.4. Porous Material Analysis

Scanning electron microscopy (SEM) measurements were performed using TM-1000 (Hitachi High-Tech Co., Minato-ku, Japan), and the surfaces of the porous bodies created through crimping were observed at 1000× magnification.

X-ray CT measurements were performed using a nano3DX (Rigaku Co., Akishima, Japan) at the Industrial Technology Institute, Miyagi Prefectural Government (ITIM, Sendai, Japan). The measurement resolution was 0.629 μm, and the reconstruction range was 1.288 mm. Pore size analysis was conducted using the form/powder analysis feature of VGSTUDIO MAX 3.5.1 (Volume Graphics Co., Charlotte, NC, USA).

### 2.5. Porosity Estimation

There are two derivations of porosities in this study: porosity obtained experimentally from bulk volume, weight, and density of the polymer, and porosity obtained from X-ray CT results. Each derivation method is described below.

The polymer used in this study had a density of 1.34 g mL^−1^ as indicated by the manufacturer’s data sheet. The sample was a cylinder with a diameter of 18 mm and a thickness of 0.6 mm or 0.5 mm, so the volume could be easily calculated. The weight of the sample was as per the weight prepared. If the sample were a perfect solid with no pores, it would be 0.204 g for a thickness of 0.6 mm and 0.170 g for a thickness of 0.5 mm, which is actually lighter. This lighter weight is caused by the pores. For example, in the case that a 0.6 mm thick sample weighs 0.083 g, it should be 0.204 g if there were no pores and 0.121 g (0.204–0.083 g) is the amount lost due to pores. Therefore, the porosity is calculated by dividing the contribution of pores by the total amount, which is 0.121 g/0.204 g = 0.59.

**Figure 3:**
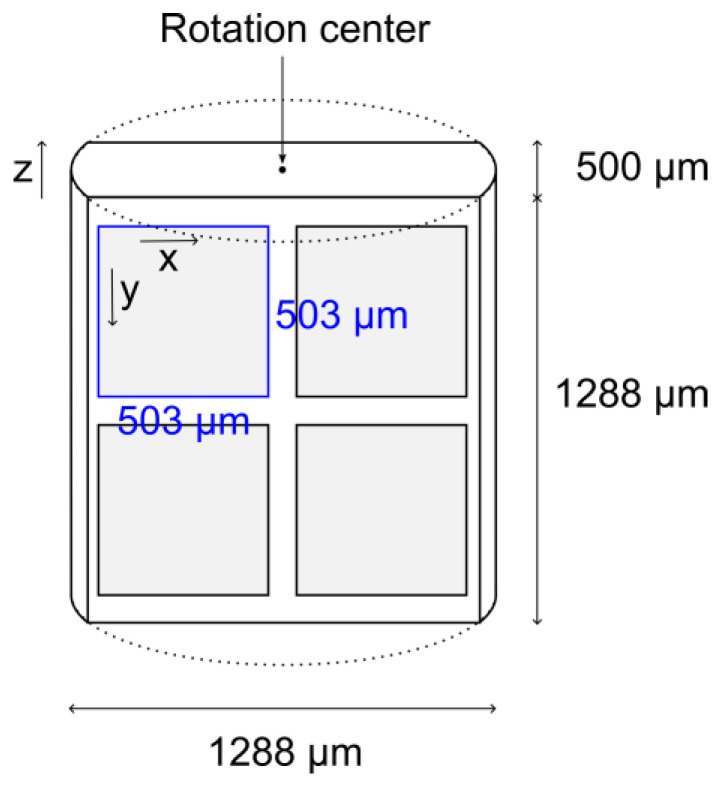
Size of reconstructed X-ray CT image and cropped area.

The first step in porosity analysis from X-ray CT is to set the analysis range. In general, the construction accuracy of X-ray CT is lower in the center of rotation. Therefore, four blocks cut to 503 μm were analyzed in the *xy*-plane direction to avoid the center of the sample (Figure 3). When analyzing porosity, the *z* direction was cut off of both surfaces, leaving a 403 μm height. This is because an improper setting of the surface of the porous material could lead to improper analysis in which the space outside of the porous material is considered pores. The size of each voxel was 0.629 μm and this analysis space has 800 voxels in the *x* and *y* directions and 640 voxels in the *z* direction. For porosity analysis, it is necessary to determine the threshold values for void and solid voxels. To determine the threshold value, a histogram of all voxel values was created, which resulted in two peaks. The small and large value peaks can be considered the porosity and the solid peak, respectively, so the intermediate value between these two peaks was used as the threshold value. Porosity was determined as the number of voxels less than the threshold value relative to the total number of voxels.

### 2.6. Pore Analysis

There are two types of pores in porous material: open pores and closed pores. The labeling of the open pore voxels uses the following algorithm.

(a) Create a cuboid consisting of voxels with 1 for solid and 0 for porosity from X-ray CT data.
(b) Place one layer of voxels with a value of 10 on the 6 surfaces of the cuboid.
(c) Scan all voxels and if the voxel contacted to the voxel with a value of 10 on the front, back, top, bottom, left, and right, has a value of 0, then that voxel is set to 10.
(d) If no voxel is changed to 10, exit; if any voxel is changed, perform (c) again.

**Figure 4:**
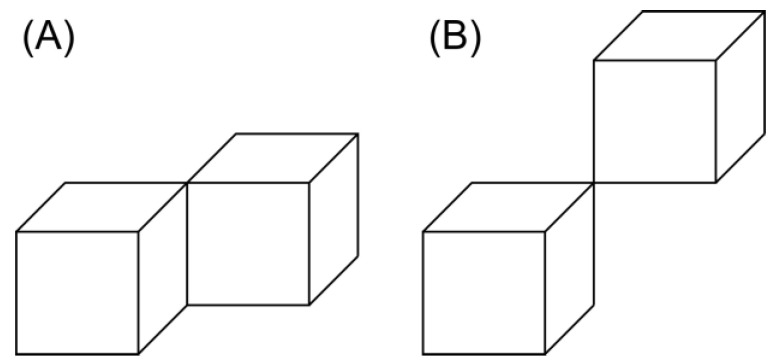
Contacted voxels at an edge or vertex in a 3-D simple cubic image system: (**A**) edge contacted and (**B**) vertex contacted.

In this paper, the surface-contacted voxels in the front, back, top, bottom, left, and right determine whether or not the hole is continuous. Therefore, contacts at edges and vertices, as shown in Figure 4, are treated as noncontacts. However, these contacts are voxels that could be judged as contacting voxels if the voxel resolution is increased. In other words, these voxels are potential connected pores. These potentially connected voxels were also counted after labeling the open pores to 10.

(e) Scan all voxels and if the voxel with 0 is in contact with a voxel with 10 on an edge (Figure 4A), the voxel with 0 is set to 20.
(f) Scan all voxels and if the voxel contacted to the voxel with 20 on the front, back, top, bottom, left, and right has a value of 0, then that voxel is set to 20.
(g) If no voxel is changed to 20, perform (h); if any voxel is changed, perform (f) again.
(h) Scan all voxels and if the voxel with 0 is in contact with a voxel with 10 on a vertex (Figure 4B), the voxel with 0 is set to 21.
(i) Scan all voxels and if the voxel contacted to the voxel with 21 on the front, back, top, bottom, left, and right, has a value of 0, then that voxel is set to 21.
(j) If no voxel is changed to 21, exit; if any voxel is changed, perform (i) again.

By performing the above operations, 20 is set for pores that are in contact at the edges and 21 is set for pores that are not in contact at the edges but at the vertices. By counting the number of voxels at 20 and 21, the number of potential open pores is estimated.

### 2.7. Tortuosity Analysis

Tortuosity, *τ*, is defined as the ratio of lengths, *L_p_* and *L*_0_, of the preferential tortuous fluid pathways and the porous media:

(1) $$\tau =\frac{L_{p}}{L_{0}}.$$

It is known that the tortuosity factor, which is the value of the square of tortuosity, has the following relationship to the self-diffusion coefficient in porous media [20]:

(2) $$\tau^{2}=\frac{D_{f}}{D_{p}},$$

where *D_p_* is the self-diffusion coefficient in the porous media and *D_f_* is the self-diffusion coefficient in free space. The tortuosity and the tortuosity factor are clearly organized by Epstein [21]. However, special attention should be paid to the definition of tortuosity since, in some research areas, the tortuosity factor is defined as tortuosity [22,23]. This self-diffusion coefficient is known to be derived from a random walk [24]. The mean square displacement, which relates to the self-diffusion coefficient, for *N* walkers is calculated as:

(3) $$\langle r^{2}\rangle =\frac{1}{N}\sum_{i=1}^{N}r_{i}^{2}.$$

In this paper, the diffusion in the *x* and *y* directions is not a concern; only that in the *z* direction is important. In this case, the tortuosity factor is calculated as:

(4) $$\tau^{2}=\frac{\langle r_{z,f}^{2}\rangle }{\langle r_{z,p}^{2}\rangle },$$

where *r_z_*_,*f*_ and *r_z_*_,*p*_ represent the z component of the random walk in free space and porous media, respectively. So, for example, if the walker starts at the origin and the plot *z_ff_*^2^ when the position becomes (*x_ff_*, *y_ff_*, *z_ff_*) after a certain number of steps in a random walk in six directions (front, back, top, bottom, left, and right), the slope of the plot is correlated with the z component of the self-diffusion coefficient.

The tortuosity is obtained by taking the square root of the tortuosity factor. The specific algorithm for determining the diffusion coefficient in a porous medium is as follows. Note that the random walk to calculate the tortuosity factor should be performed for the open pores so that the voxels of the open pores are first labeled with the operation described in Section 2.6.

(a) Label the voxels in the aperture pore as 10 using the labeling operation for the aperture pore in Section 2.6.
(b) Then, exclude 200 voxels vertically, horizontally, vertically, front to back, and left to right, and randomly designate a voxel in the center area with a value of 10 as the starting point.
(c) Perform 10,000 random walks and record the average value.

In a random walk with obstacles, the direction of the walker is determined by a random number. If the walker can move, the walker moves forward, backward, up, down, left, or right. If not, the walker stays where it is. In the case of free space, there are no obstacles, so the walker will always move in the direction specified by a random number.

### 2.8. Pore Size Analysis

In this study, two derivations of pore size are obtained from the permeability test and the X-ray CT results. The derivation method for each is described below.

Derivation of pore size from liquid permeation is obtained by analysis of flow in the pores as flow in a capillary tube. A liquid flow rate *Q* through a circular capillary tube is described by the Hagen–Poiseuille equation as follows [25]:

(5) $$Q=\frac{\pi d^{4}∆p}{{128\mu L}_{0}},$$

where *d* is the capillary diameter, Δ*p* is the pressure drop along the capillary, *μ* is fluid viscosity, and *L*_0_ is length of the capillary tube. Dividing *Q* by the cross-sectional area and converting it to velocity *v*_0_, the above equation becomes:

(6) $$v_{0}=\frac{d^{2}∆p}{{32\mu L}_{0}}.$$

When porosity is *α*, the actual flow is only in the pores, resulting in a velocity replaced with *v*_0_/*α*, which transforms the above equation into

(7) $$v_{0}=\frac{d^{2}∆p\alpha }{{32\mu L}_{0}}.$$

Furthermore, if there is a bend in the flow path (*τ* = *L_p_*/*L*_0_), the velocity becomes *τ v*_0_ in addition to the effect that the length of capillary *L*_0_ should be replaced with *τ L*_0_. Considering both effects, Equation (7) becomes

(8) $$v_{0}=\frac{d^{2}∆p\alpha }{{32\mu L}_{0}\tau^{2}}.$$

From this equation, the pore diameter *d* of the porous media can be evaluated by the following equation.

(9) $$d=\sqrt{\frac{{32v}_{0}{\mu L}_{0}\tau^{2}}{∆p\alpha }}.$$

To derive pore size from X-ray CT, spatial analysis was performed using the form/powder analysis in VGStudio max 3.5.1. This analysis outputs a list of pore area and pore volume. Assuming a cylinder for each pore, the diameter and length of a cylinder that satisfy the output pore area and pore volume were derived using the Newton method with the equivalent sphere diameter multiplied by the sphericity as the initial value. The diameter of the cylinder was used as the pore diameter. The reason for setting the initial values in this way is to obtain a short diameter solution of two solutions. There are two solutions that satisfy the specified pore area and pore volume: a short diameter and long length, and a flat disk-shaped cylinder with a large diameter and extremely short length. The log differential pore volume distribution was calculated by integrating the obtained pore diameter and pore volume.

## 3. Results and Discussion

**Figure 5:**
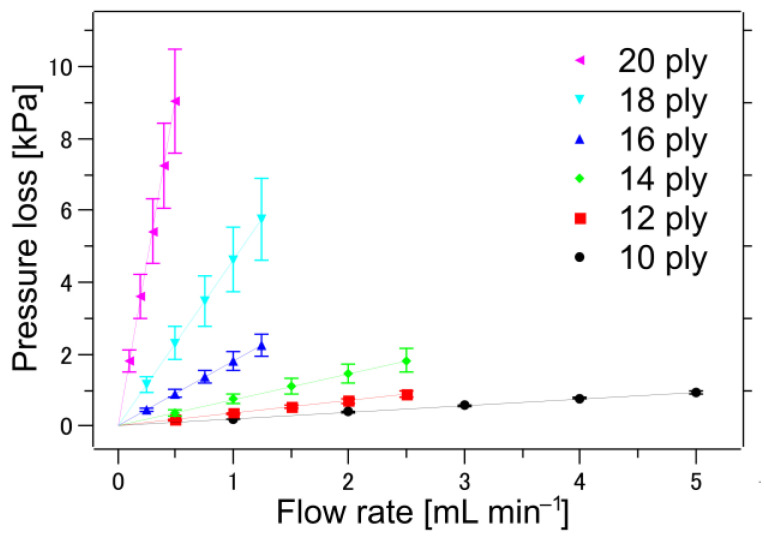
Pressure loss as function of flow rate for various 0.6 mm-thick CAPC-made samples consisting of nonwoven fabric and different numbers of sheets. The straight lines passing through the graph origins represent the fitting of the experimental results. Error bars represent the standard deviations of the five experiments.

A 10 wt% ethanol aqueous solution was used to ensure sample wettability during the flow test. Initially, a water flow test with pure water was performed, but unwetted portions remained in the sample. Vacuuming was also conducted, but the generation of air bubbles could not be sufficiently prevented, and stable measurement results could not be obtained. The measurement of 10 wt% ethanol solution resulted in stable results, and a pressure loss measured with 10 wt% ethanol solution was one order of magnitude smaller than that measured with water, i.e., it is more permeable. The experimental results for the 10 wt% ethanol aqueous solution are shown in Figure 5. Here, the measurements were conducted on five samples, and the average values of the experimental results were plotted with error bars for the standard deviation. We found that the results of the liquid flow test for each sample were quite linear, and the variation shown in Figure 5 is because of sample variation. The nonwovens used in this study were made on a prototype machine that can be manufactured up to a 500 mm width. Therefore, they are more nonuniform than industrially mass-produced nonwoven fabrics. If a more homogeneous nonwoven fabric is used, the variation can be further reduced.

**Figure 6:**
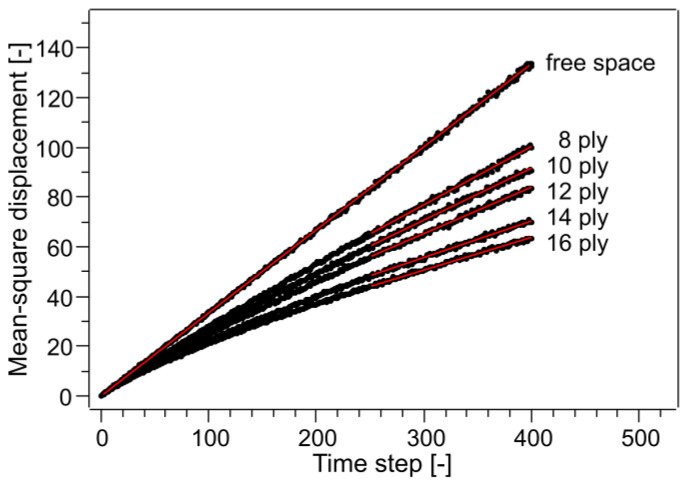
Mean-square displacement of the z component of the lattice walk averaged over 40,000 walkers. The data of stacked samples are an average of the four blocks (10,000 walkers for each block) shown in Figure 3. Red lines are the least-square fitting of a certain range.

**Figure 7:**
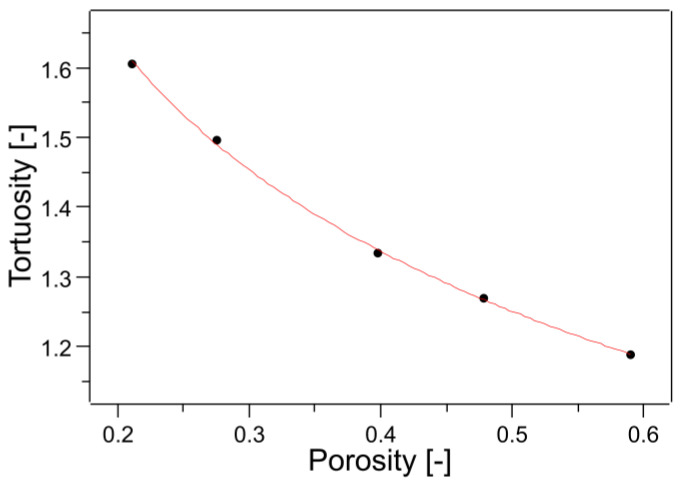
Tortuosity as a function of porosity. The red line is the least square fitting using Equation (10) with *α_c_* equal to zero.

To obtain the pore diameter of a porous material with this equation, an estimate of the tortuosity is necessary. Koponen et al. proposed the following equation with a two-dimensional estimate of the tortuosity [26]:

(10) $$\tau =1+A\frac{1-\alpha }{{\left(\alpha -\alpha_{c}\right)}^{m}},$$

where *α_c_* is critical porosity and *A* and *m* are fitting parameters determined to be 0.65 and 0.19, respectively. Critical porosity is the porosity at which there are no through holes and the tortuosity becomes infinite. Since a critical porosity value of 0.33 has been proposed, this equation is not applicable to the CAPC porous material, which is able to permeate liquid with a porosity of 0.2. Since 3D structural data were obtained by X-ray CT, the tortuosity was estimated based on the data. The results of the self-diffusion coefficient obtained by random walk are shown in Figure 6. The least-square fitting slope in a free-space slope of 0.3335 is close to the value of one-third. This is because there is movement in the *x* and *y* directions in a 3D random walk, so the movement in the *z* direction is one-third in probability. The random walk in free space was linear from 1 to 400 steps, but the random walk in porous media required a certain number of steps before stabilization. This indicates that there is a necessary number of steps before the shape of the porous media can be recognized. For actual analysis, the portion of the graph that was linear from 251 to 400 was fitted and the slope was used as the self-diffusion coefficient. The self-diffusion coefficient of free space divided by the self-diffusion coefficient of porosity is the tortuosity factor and the square root is the tortuosity. A plot of tortuosity versus porosity is shown in Figure 7. Error bars are not shown in Figure 7 because the errors obtained from least-squares fitting fall within the range of the markers. Porosity was calculated simply by counting the voxels of voids and solids; Table 1 shows the porosities calculated from the X-ray CT images and those from polymer volumes and densities. The values are roughly close. The imperfect agreement in porosity could be due to the way the threshold value was determined or insufficient resolution (0.629 μm) of the X-ray CT. This suggests that future analysis may show a 10–20% difference between the actual sample and the X-ray CT.

As a rapid increase due to critical porosity was not observed in Figure 7, fitting was performed using Equation (10) in the case of *α_c_* equal to zero. The fitting results are drawn in Figure 7 and are good. The description, including the fitting parameters, is

(11) $$\tau =1+0.355\frac{1-\alpha }{\alpha^{0.501}}.$$

This means that the tortuosity of the CAPC porous material was successfully obtained as a function of porosity. Since the tortuosity plays an important role in analyzing the flow and diffusion in the porous media, this tortuosity formula is very useful for the design of these components. In a previous study, a model using critical porosity was proposed for a porous material with randomly stacked fibers in the transverse direction, which agrees well with the experimental results of the current study for air permeability [27]. The tortuosity is known to take different values depending on the fiber orientation [28], and for a porous material with randomly stacked fibers in the transverse direction, the tortuosity of nonwoven glass fiber filter is 6.4 (41.6 as tortuosity factor) when the porosity is 0.86, showing a significant increase with a decrease in porosity [29]. In comparison, the tortuosity in this study is low and maintains permeability even at low porosity.

Table 2 summarizes all experimental results, including the pore sizes estimated using Equation (9). The pressure loss at 1 mL min^−1^ is the average of the slopes of five experiments (the errors are standard deviations). The viscosity of the aqueous ethanol solution at 20 °C *μ* was set to 1.366 × 10^−3^ Pa s, which was calculated at 20 °C using a formula from the literature [30]. The porosity values in Table 2 were calculated from the sample volume and weight. Tortuosity, *τ*, was calculated by Equation (11). When the diameter of the membrane’s flow area is 8.73 mm, *v*_0_ is 2.79 × 10^−4^ m s^−1^ at a flow rate of 1 mL min^−1^. The sample thickness, *L*_0_, was 6 × 10^−4^ m. Table 2 shows that the smaller the porosity, the smaller the pore size, which is consistent with a previous study [9].

**Figure 8:**
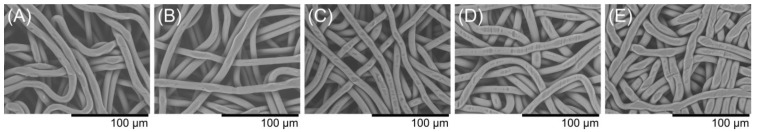
Scanning electron microscopy images of sample surfaces. (**A**) 8-ply laminated product, (**B**) 10-ply laminated product, (**C**) 12-ply laminated product, (**D**) 14-ply laminated product, (**E**) 16-ply laminated product.

**Figure 9:**
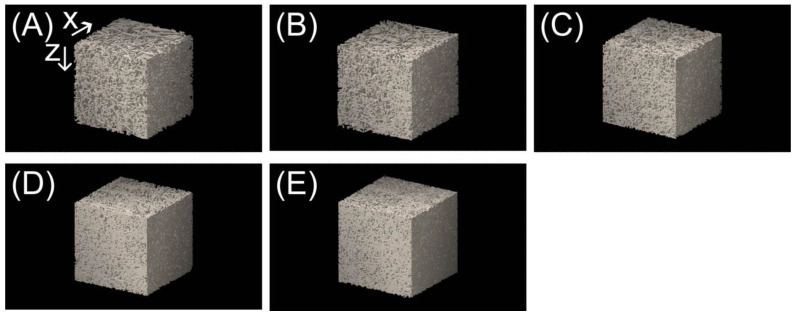
X-ray computed tomography images. The sample thickness in the *z* direction is 0.500 mm; the width in the *x* and *y* directions is 0.503 mm. (**A**) 8-ply laminated product, (**B**) 10-ply laminated product, (**C**) 12-ply laminated product, (**D**) 14-ply laminated product, (**E**) 16-ply laminated product.

**Figure 10:**
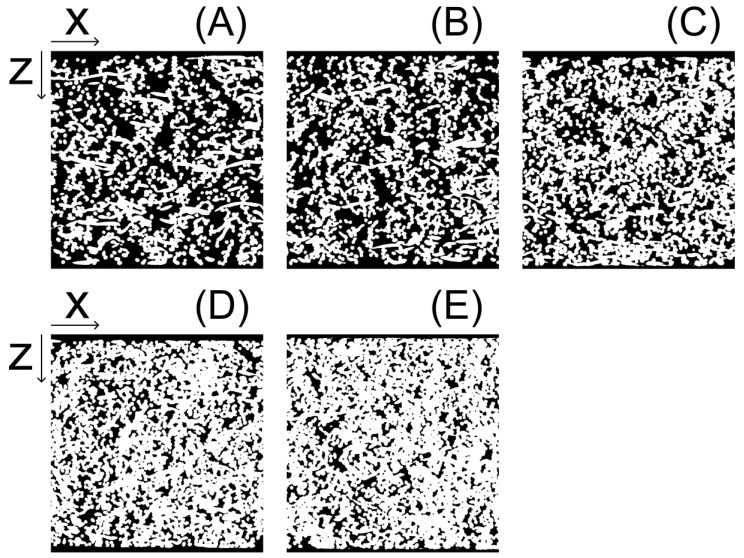
Cross section of X-ray computed tomography images. The images are binarized to make it clear whether or not the fibers are in contact: (**A**) 8-ply laminated product, (**B**) 10-ply laminated product, (**C**) 12-ply laminated product, (**D**) 14-ply laminated product, and (**E**) 16-ply laminated product.

Figure 8 shows SEM images of the surfaces of the samples, adjusted to a 0.5 mm thickness. Foaming of polymers has been studied using CO_2_ under supercritical conditions [31]. In this study the treatment was performed at room temperature (23 °C), below the critical point (31.1 °C), for a short time (10 s) and no foam traces were observed on the fiber surfaces. Foaming occurs in competition with the desorption of CO_2_ from the polymer, which requires high CO_2_ solubility and a large pressure differential between the inside and outside of the polymer. Typically, in the foaming process, CO_2_ is dissolved at a high pressure over a long period, followed by rapid depressurization. In this study, dissolution was conducted for a short period at a pressure less than the critical pressure, and depressurization was slowly conducted through a metering valve for the first 30 s. The large surface area (due to the small diameter of the fibers) also facilitates the desorption of CO_2_, which is not conducive to foaming. Figure 9 shows the X-ray CT measurements of a sample prepared under the same conditions as in Figure 8. The sample thickness in the *z* direction in Figure 9 is 0.5 mm. As seen in Figure 8 and Figure 9, the porosity and pore size decrease as the number of nonwoven fabric layers increases. Cross-sectional analysis can be performed using the X-ray CT images, and the connection of the pores from the front surface to the back surface of the filter with tortuosity is clear (vertical surfaces in Figure 9). The cross section can be determined from Figure 9, but it is difficult to understand. Therefore, a slice image of the center part is shown in Figure 10. The first noteworthy point is that a porous material is generated in which the boundary of nonwoven fabrics is not recognizable at all, even though it was originally created by crimping nonwoven fabric-laminated products. The number of adhesion points between fibers in Figure 10 were counted for 8, 10, 12, 14, and 16-ply laminated products to be 743, 854, 1091, 1226, and 1346, respectively. The number of adhesion points monotonically increased as the number of laminated sheets increased. This is the same phenomenon as the cross-sectional analysis model proposed in a previous study of adhesive strength [32]. Note that as the number of laminated sheets increases, the connection from the surface to the back cannot be evaluated from a single cross section and it is necessary to determine whether or not the connection is three dimensional. Unlike heat-pressed samples, these samples have no molten polymer clumps and have voids throughout; this structure is ideal for filters.

**Figure 11:**
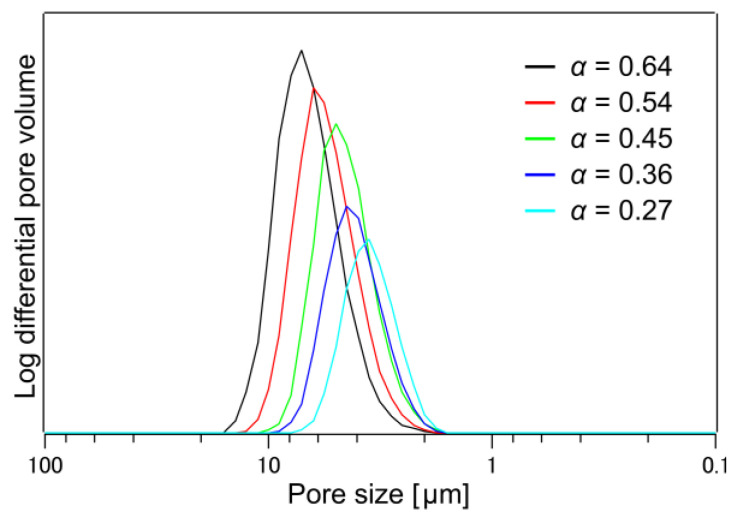
Pore size distribution evaluated via X-ray computed tomography. The indicated porosity *α* is calculated from X-ray computed tomography images.

The results of the log differential pore volume distribution estimated from the X-ray CT structural analysis are shown in Figure 11. Pore size analysis was performed for the vacant sites using the form/powder analysis feature of VGSTUDIO MAX 3.5.1. The output number of pores was approximately 41,000 for the 8-ply sample (total of four blocks) and 106,000 for the 16-ply sample (total of four blocks). Similar to the pore size results from the permeation test (Table 2), the pore size decreases with decreasing porosity and the maximum pore size shown in Figure 11 is also somewhat similar.

Table 3 summarizes the porosity calculated from the X-ray CT and the percentage of open pores. The ratio of open pores is more than 99% when the porosity is greater than 0.28. The case with a porosity of 0.21 also maintains a high percentage of open pores of 98.5%. In this case, 12% of the pores determined to be closed pores were in contact at the edges and 2% at the vertices, which might be potential open pores. The reason for this high open-pore percentage is thought to be related to the CAPC manufacturing method: CO_2_ is trapped in the polymer at about 6 MPa during the adhesion process and is released to the atmosphere under a pressed condition. In other words, there is an exhaust path for CO_2_ to exit, which should be a through hole. This high open-pore ratio even at low porosity is consistent with the fact that the tortuosity could be fitted well with an equation that does not have critical porosity. This high ratio of open pores can be said to be the optimum structure for applications such as fluid permeation and drug release, and I propose future development of components for such applications.

## 4. Conclusions

Liquid permeation performance was evaluated by conducting a permeation test with a 10 wt% ethanol solution. The three-dimensional structure of CO_2_-assisted polymer compression (CAPC) porous material was analyzed by X-ray computed tomography (X-ray CT) to evaluate the tortuosity and the percentage of open pores to all pores. The following results were obtained.

- The pore diameters estimated from the liquid permeation test and obtained from X-ray CT images were roughly in agreement.
- The cross section from X-ray CT data showed formation of a completely integrated porous material with no visible seams between the nonwoven fabrics.
- X-ray CT structural analysis showed that tortuosity of the CAPC porous material can be estimated using the following equation: 1 + 0.355 (1 − α)/α^0.501^.
- The percentage of open pores of the CAPC porous material was very high (98.5%) at a porosity of 0.21.
- The high open-pore ratio may be a result of the CAPC manufacturing process, wherein CO_2_ trapped under high pressure is exhausted.

These results indicate that CAPC porous materials are suitable for applications, such as fluid filters and sustained-release drug tablets.

## Figures and Tables

**Table 1:** Sample properties for X-ray computed tomography and analysis results.

Number of Sheets [-]	Weight [g]	Thickness [mm]	Porosity Calculated from Polymer Density [-]	Porosity Calculated from X-ray CT Image [-]
8	0.062	0.500	0.64	0.59
10	0.078	0.500	0.54	0.48
12	0.093	0.500	0.45	0.40
14	0.109	0.500	0.36	0.28
16	0.125	0.500	0.27	0.21

**Table 2:** Experimental results of ethanol solution permeability of CO_2_-assisted polymer compression products and pore diameter estimated by Equation (9).

Number of Sheets [-]	Weight [g]	Thickness [mm]	Porosity [-]	Pressure Loss at 1 mL min^−1^ (ΔP) [kPa] *	Tortuosity [-]	Pore Diameter [μm]
10	0.083	0.600	0.59	0.19 ± 0.01	1.19	9.5
12	0.099	0.600	0.52	0.35 ± 0.03	1.24	7.9
14	0.116	0.600	0.43	0.73 ± 0.14	1.31	6.3
16	0.132	0.600	0.35	1.8 ± 0.2	1.36	4.7
18	0.149	0.600	0.27	4.6 ± 0.9	1.50	3.7
20	0.165	0.600	0.19	18 ± 3	1.65	2.4

* Error indicates standard deviation.

**Table 3:** Sample properties for X-ray CT and analysis results.

Number of Sheets [-]	Porosity Calculated from X-ray CT Image [-]	Ratio of Open Pore [%]
8	0.59	100
10	0.48	100
12	0.40	99.9
14	0.28	99.5
16	0.21	98.5

## Data Availability

All data generated or analyzed during this study are included in this published article.
